# Effect of rapid maxillary expansion on sleep apnea-hypopnea syndrome in growing patients. A meta-analysis

**DOI:** 10.4317/jced.55974

**Published:** 2019-08-01

**Authors:** Ana-Matilde Sánchez-Súcar, Francisco-de Borja Sánchez-Súcar, José-Manuel Almerich-Silla, Vanessa Paredes-Gallardo, José-María Montiel-Company, Verónica García-Sanz, Carlos Bellot-Arcís

**Affiliations:** 1 Doctorate student, Department of Stomatology, Faculty of Medicine and Dentistry, University of Valencia; 2 Doctorate student, Department of Stomatology, Faculty of Medicine and Dentistry, University of CEU Cardenal Herrera Valencia; 3 Tenured Lecturer, Department of Stomatology, Faculty of Medicine and Dentistry, University of Valencia; 4 Teaching Assistant, Department of Stomatology, Faculty of Medicine and Dentistry, University of Valencia; 5 Associate Professor, Department of Stomatology, Faculty of Medicine and Dentistry, University of Valencia; 6 Assistant Professor, Department of Stomatology, Faculty of Medicine and Dentistry, University of Valencia

## Abstract

**Background:**

Changes produced in the upper airway after rapid maxillary expansion makes this procedure a therapeutic option for treating sleep apnea-hypopnea syndrome (SAHS) in children. The objective of this systematic review and meta-analysis was to analyze the evidence available for the effects of rapid maxillary expansion (RME) on SAHS, analyzing changes produced in oximetric variables: apnea-hypopnea index (AHI); oxygen saturation (SO2); sleep efficiency (SE), total sleep time (TST), percentage of rapid eye movement (REM) phase; and arousal index (AI).

**Material and Methods:**

An electronic search was conducted in the PubMed, Scopus, Embase, and Cochrane databases, and in grey literature (Opengrey). No limit was placed on publication date or language. Inclusion criteria were: patients in growth with sleep apnea who underwent rapid maxillary expansion with oximetric values registered before and after treatment. Articles with patient sample sizes <10 were excluded. Ten articles were included for qualitative synthesis and nine for meta-analysis (eliminating one observational study).

**Results:**

AHI values underwent a mean reduction of 5.79 events/hour (CI -95% 9.06 to 2.5); an increase in mean oxygen saturation of 2.54 % (CI-95% -0.28 to 4.80, 6.7 %); a reduction in AI of 2.17 events/hour (CI-95% -5.25 to -0.582); an increase in REM phase of 1.20 % (CI-95% 1.02 to 1.38); and an increase in SE of 0.961% (CI-95% -1.574 to 3.495).

**Conclusions:**

RME would appear efficient for treating slight or moderate SAHS, as indicated by improvement in oximetric parameters; it may be effective as coadjuvant therapy to adenotonsillectomy in severe cases of children with maxillary compression.

** Key words:**Rapid maxillary expansion, obstructive sleep apnea.

## Introduction

According to the American Academy of Sleep Medicine, sleep apnea-hypopnea syndrome (SAHS) in children can be defined as multiple episodes in which obstruction of the upper airway (UA) is produced, impeding respiration during sleep. SAHS produces reductions in partial oxygen saturation, which induces a micro-awakening to reestablish normal oxygen flow (1). These events interrupt the normal structure of sleep and impede its reparative function (2). This can trigger excessive daytime sleepiness, underachievement at school, and in severe cases, neuropsychiatric disorders and a risk of cardiovascular disorders; all these problems impact on the patient’s quality of life (3).

SAHS has a prevalence of 1-10% in the pediatric population (3), the most common cause of SAHS being amygdala hypertrophy, although its etiology is usually due to a combination of diverse anatomical and functional factors that produce the collapse of the UA (3). The most common clinical manifestations are habitual snoring, mouth breathing, increased respiratory effort (2), night sweats, abnormal sleeping positions (such as hyperextension of the neck), restlessness, and nocturnal enuresis (3).

According to the American Academy of Pediatrics (4), polysomnography is the diagnostic method of choice for children, and it is accepted that an apnea-hypopnea index AHI (number of apnea and hypopnea events per hour of sleep) of between 1 and 3 constitutes the threshold between normal and abnormal sleep (2,4).

The treatment of choice for children is adenotonsillectomy (2,4) with 78% efficacy (3); nevertheless, it has not been shown that this resolves SAHS completely (5,6) and it is an invasive form of treatment for the child patient. The second option is continuous positive airway pressure (CPAP) (3,4). This palliative treatment causes discomfort and most patients do not become accustomed to it, which makes them uncooperative (5).

High prevalence of SAHS combined with a low rate of diagnosis and the serious impact on the quality of life of the child patient make it a health concern that the dentist should look out for in routine clinical practice. There is also a need to investigate new and more effective alternative therapies for treating this syndrome. In this context, and in light of the apparently promising results obtained in research, RME would appear to offer a SAHS treatment that is more effective and less invasive.

The aim of this study was to conduct a systematic literature review and meta-analysis to determine the effect of RME on SAHS and analyze its effects on the apnea-hypopnea index (AHI), oxygen saturation (SO2), sleep efficiency (SE), total sleep time (TST), percentage of rapid eye movement phase (REM) and arousal index (AI).

## Material and Methods

This systematic literature review was conducted following PRISMA guidelines (Preferred Reporting Items for Systematic Reviews and Meta-Analyses) (7) and was registered in the PRISMA database (PROSPERO) (reference No. CRD42017037378).

-PICO question

The review set out to answer the following PICO (patient, intervention, comparison, outcome) question: In patients in growth with sleep apnea-hypopnea syndrome, does rapid maxillary expansion improve clinical variables or variables registered in polysomnography? 

-Inclusion and exclusion criteria 

Both “articles” and “articles in press” were included: randomized clinical trials, cohort studies, and case/control studies (both retrospective and prospective). No restriction was placed on the publication date or the language in which articles were published. Inclusion criteria were: studies of patients in growth with SAHS who underwent RME, which registered oximetric values by means of polysomnography. Studies with patients samples <10 were excluded.

-Search strategy

Sources of information

To identify all studies of potential relevance, regardless of the language in which they were published, a rigorous electronic search was conducted in the PubMed, Scopus, Embase, and Cochrane databases. An electronic search was also conducted for grey literature in the Opengrey database. In some case, the authors were contacted by e-mail in order to request missing information. The search was completed manually, reviewing the bibliographies of the articles identified to locate further works that might have been missed in the initial search. The systematic review and meta-analysis was conducted in October 2017.

Search terms 

A list of search terms was formulated consisting of MeSH terms (Medical Subject Heading), as well as non-indexed terms that might identify relevant texts. Boolean operators (“OR” and “AND”) were used to link search terms (both MeSH/ and non indexed). Based on the PICO question, the following search strategy was used: ((((((((((“Sleep Apnea, Obstructive”[Mesh]) OR “Sleep Apnea Syndromes”[Mesh]) OR “Snoring”[Mesh]) OR “Sleep Disorders, Intrinsic”[Mesh]) OR Breathing) OR OSA) OR “respiration”[MeSH Terms])) AND ((((“disease”[MeSH Terms]) OR Disorders) OR “Palatal Expansion Technique”[Mesh]) OR “Child”[Mesh])) AND ((((“palatal expansion technique”[MeSH Terms]) OR Maxillary expansion) OR RME) OR Rapid maxillary expansion)) AND (((OSA improvement) OR sleep apnea syndrome healing) OR obstructive apnea improvement).

Study selection

Two independent reviewers (AMS-S and CB-A) systematically and independently assessed both the titles and the abstracts of the articles identified. In case of any disagreement, a third reviewer was consulted (JMM-C). If an abstract failed to provide sufficient information to reach a decision, the full text was read before deciding whether or not to include the article in analysis. Afterwards, the complete articles were read and the reasons for excluding articles were registered.

Study data

The following variables were registered for each article: author and year of publication, sample size, demographic variables (sex and mean age), type of study, patients lost during the study, AHI, SO2, SE, AI, TST, and percentage of REM phase sleep.

-Quality assessment 

The quality of the studies was evaluated by the same researchers independently using the CONSORT quality criteria developed by Mattos *et al.* (8) for experimental studies. In cases of discrepancy between the two researchers, consensus was reached and in case of any doubt, the third researcher was consulted.

-Variables and synthesis of results 

Mean values and initial and final confidence intervals were registered for the following variables: AHI, SO2, SE, TST, percentage of REM, and AI.

-Statistical analysis 

For quantitative synthesis, the differences between mean values were calculated and the confidence intervals (CI) between initial and final states. Heterogeneity was assessed with the Q test and the I2 test. Heterogeneity was considered to exist when the Q test p-value was lower than 0.1. The combined random effects method was used to estimate mean differences. Publication bias was assessed by means of funnel plots and Rosenthal’s fail-safe number. Meta-analysis was performed using Comprehensive Meta-analysis Software V 3.0.

## Results

-Article selection process and flow diagram 

The initial electronic search identified 149 references related to changes produced in the upper airway after RME, of which 76 appeared in the Pubmed database, 45 in Embase, 22 in Scopus and 6 in Cochrane; one article was located in grey literature. Forty-two articles were duplications leaving a total of 108. A further 75 were excluded after reading the titles and abstracts, as they were unrelated to the PICO question. A manual search was conducted among the bibliographies of the remaining 33 articles, identifying four more works. After reading the complete text, 27 were eliminated for the following reasons: three investigated adults or young adults; in one the sample did not meet the SAHS criterion, one assayed maxillary advancement, one lacked tables (the author was contacted but no reply received), two investigated malformation syndromes, five used repeated patient samples, one performed adenotonsillectomy simultaneously with RME, three because they did not provide sufficient study variable data, four did not carry out polysomnography, three were clinical case reports, two were literature reviews, and one was subject to an erratum and had been eliminated by PubMed.

**Figure 1:**
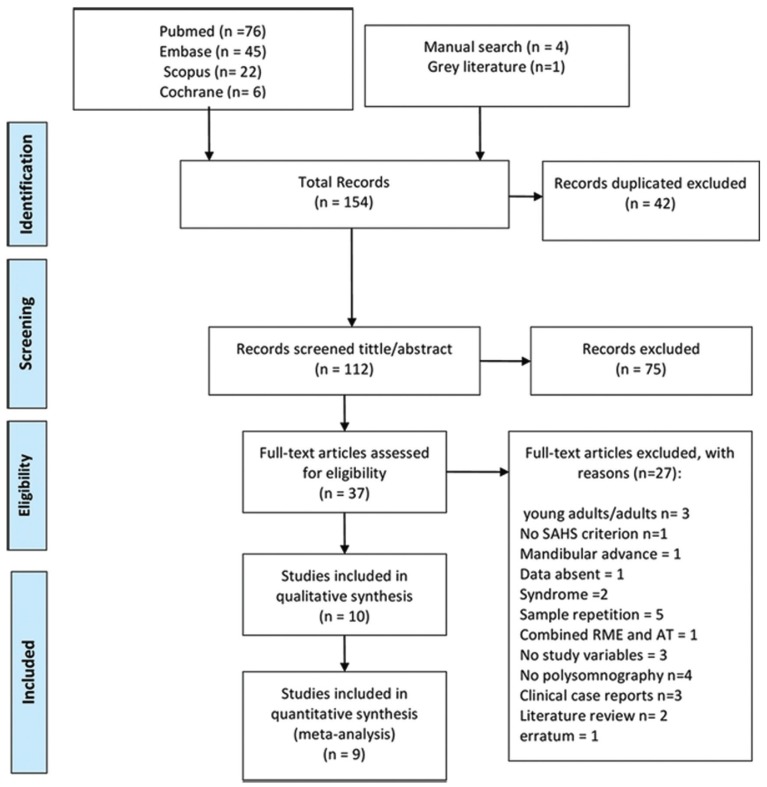
Flow diagram.

Finally, 10 articles fulfilled the inclusion criteria and were included for qualitative analysis and nine for quantitative synthesis (meta-analysis), as one article was an observational study (9). The selection process is illustrated as a PRISMA flow diagram (Fig. 1).

-Study characteristics 

The studies included for analysis all had samples of at least 10 patients. All indicated that the sample included patients with maxillary compression, and stated the patients’ mean age and sex. All patients were growing children with an average age of around 8 years.

Of the 10 studies, three had control groups, six had no control subjects and were not randomized, and one was an observational study (9) (and so excluded from quantitative analysis). With regard to the control groups, one group received no treatment (10), another control group underwent adenotonsillectomy (AT) (11), while in the remaining randomized study both groups underwent AM and RME but in different order (12).

Most of the articles were of moderate quality according to CONSORT criteria modified by Mattos *et al.* (8) ( Table 1).

**Table 1:**
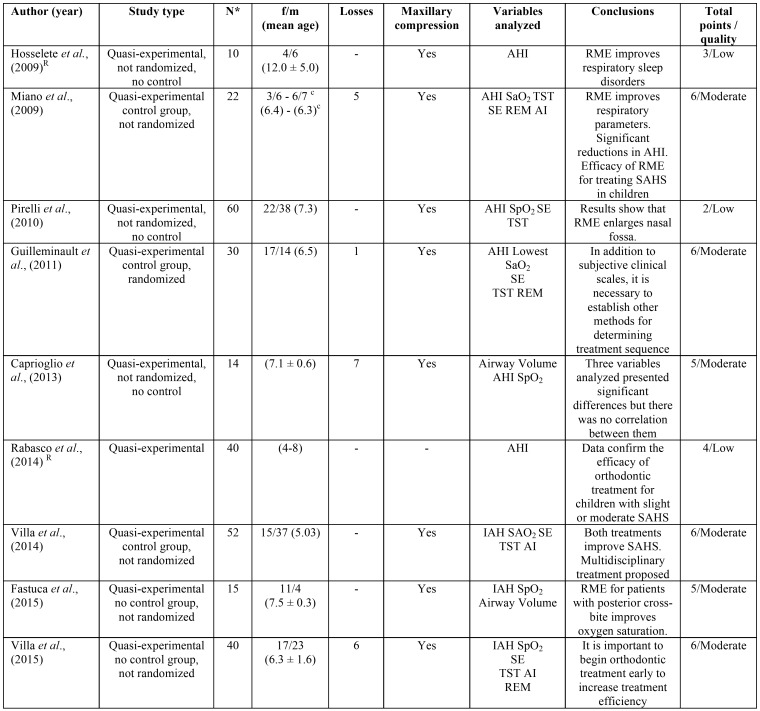
Characteristics of studies analyzed and CONSORT quality criteria developed by Mattos *et al.*, (2011).

-Qualitative synthesis 

All studies observed improvements in apnea-hypopnea index (AHI) values after RME (10-15), with the exception of one work by Miano *et al.* (10). Guilleminault *et al.*(12) investigated the effect of performing adenotonsillectomy first or RME first in the treatment sequence, finding that after the first treatment phase (T1) AHI did not reach normal values – a residual component of the syndrome remained – although the improvement was statistically significant. At the end of the second phase (T2), having performed both AT surgery and RME, normal AHI values were obtained, regardless of the order in which the treatments wee applied. In the two works of better quality, a residual component also remained after treatment, which also disappeared after combined therapy with AT and RME. Twelve years after RME, AHI remained stable and within the range of normality (9).

Oxygen saturation (S02) was measured in five studies, finding a significant decrease in hypoxia values in three of them (<92%), and reaching normal values after RME (14-16). But two works did not observe statistically significant changes in SO2 (10,11). Desaturation time (duration of saturation <92% within total sleep time) decreased and remained stable 12 years after treatment (9).

Sleep efficiency (SE) was measured in four works, obtained by dividing total sleep time between the hours the child spent in bed and the real duration of reparative sleep. In three this was found to increase after treatment but without statistical significance (10,11,16). Miano *et al.* (10) did not find significant differences in SE between the group with SAHS and the control group either before or after treatment. Pirelli et al. found that sleep efficiency remained stable 12 years after treatment with RME (9).

Total sleep time (TST) was measured in four studies (10-12,16). Two obtained significant differences (11,12) with an increase in total hours of sleep after treatment. Guilleminault *et al.* found that patients slept for longer at the end of the first treatment phase (12), whether this was RME or AM, and continued to increase after the second phase, regardless of the order of treatment. Miano *et al.* (10) found significant differences between the control group and SAHS patients before treatment, the control group sleeping longer, but did not find differences in the SAHS group before and after treatment.

The percentage of rapid eye movement (REM) sleep was measured in three studies. In two, no significant differences were observed (10,16), and in the randomized case/control study by Guilleminault *et al.* (12) a significant increase was obtained after both treatment phases (T1 and T2), regardless of the order of treatment (RME or AT performed first).

The arousal index (AI) is the number of micro-awakenings divided by TST, which was measured in two studies. In Villa *et al.* (11) the AI did not decrease significantly, while Miano *et al.* (10) found a significant difference between SAHS and control patients before treatment, but AI did not decrease significantly in the SAHS group as a result of treatment by RME.

 Table 1 shows details of the studies selected, registering the type of study, sample size, losses, demographic variables, oximetric variables (AHI, SO2, SE, TST, percentage of REM, and AI).

-Quantitative synthesis 

Oximetric changes 

**Figure 2:**
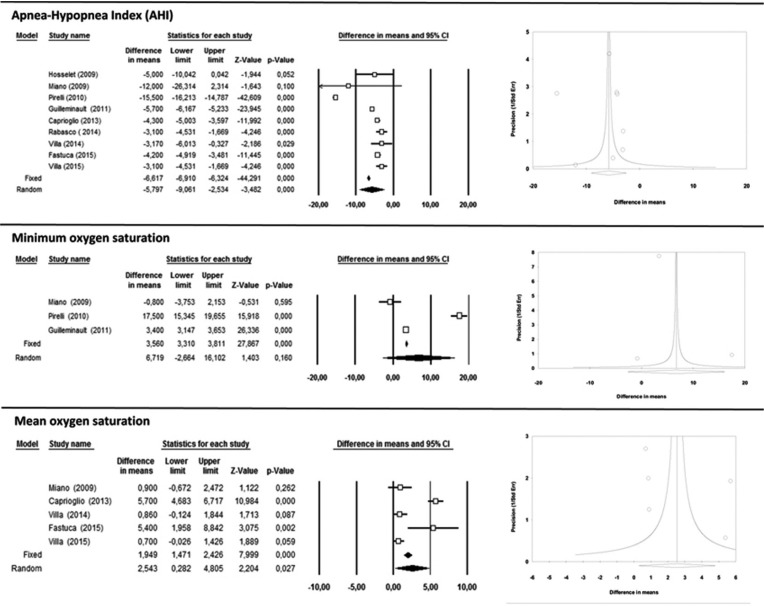
Apnea-Hypopnea Index (AHI), Minimum oxygen saturation and Mean oxygen saturation forest plots and funnel plots.

The apnea-hypopnea index (AHI) (Fig. 2) was registered in all the studies selected and all found significant decreases as a result of treatment by RME. The random effects model obtained a difference in means of 5.79 events/hour with a 95% CI (9.06 to 2.5) considered to present statistical significance (*p*<0.001), and showing high heterogeneity (I2> 75%) of 98.9% (Q=749.3; *p*=0.000).

With regard to oxygen saturation (SO2), minimum saturation was measured in three of the studies (10,12,13), while the others registered mean oxygen saturation (10,11,14-16). The random effects models shown in Figure 2 estimate a difference in means of 6.7% with a 95% CI (-2.7 to 16.1), which was not statistically significant for minimum saturation, while for mean saturation an increase of 2.5% was obtained with a 95% CI (0.28 to 4.80), which was statistically significant (*p*<0.027). Heterogeneity was high (I2> 75%) for both minimum oxygen saturation and mean saturation: I2=98.8% and I2=98% respectively.

-Sleep variables

**Figure 3:**
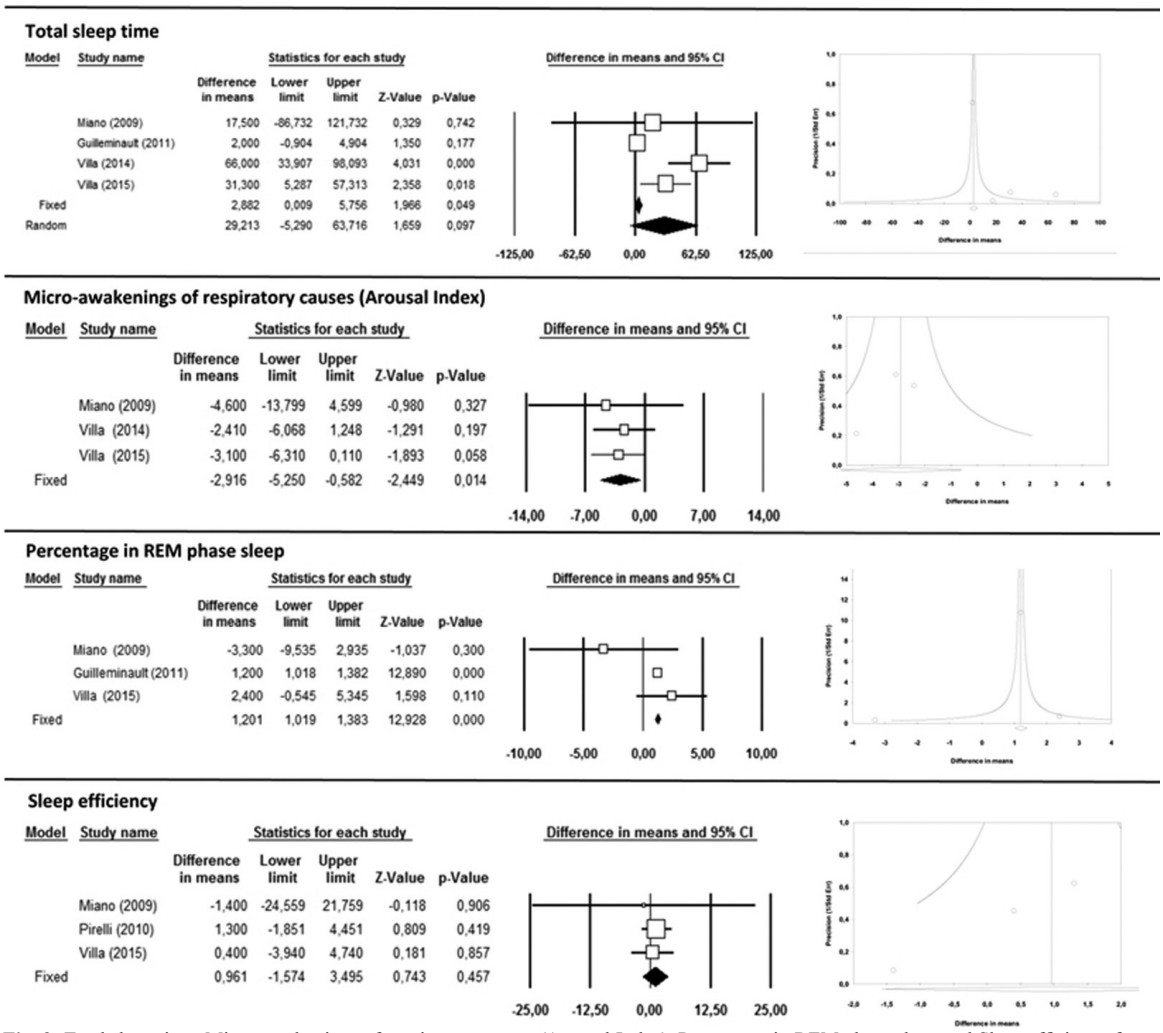
Total sleep time, Micro-awakenings of respiratory causes (Arousal Index), Percentage in REM phase sleep and Sleep efficiency forest plots and funnel plots.

Total sleep time (TST) showed a difference in means of 29.2 minutes with a 95% CI (-5.29 to 63.7), which was not statistically significant (Fig. 3). For measurements of micro-awakenings of respiratory causes (arousal index, AI), a decrease of 2.17 events per hour with a 95% CI (-5.25 to –0.58) showed statistical significance (*p*<0.001) (Fig. 3). The percentage of REM phase sleep underwent significant increases in all studies, with an increase in REM sleep of 1.2% after RME with a 95% CI (1.02 to 1.38) (*p*<0.001) (Fig. 3). Sleep efficiency (SE) underwent a difference in mean values of 0.96% with a 95% CI (-1.57 to 3.5) with statistical significance (Fig. 3).

Among all the sleep variables measured, TST presented the highest heterogeneity of results (I2= 84,9%), while the AI, REM and SE showed low heterogeneity (I2= 0%, I2=24.2%, I2= 0%, respectively).

-Publication bias 

In general, meta-analysis funnel plots (Figs. 2,3) show little symmetry between the studies analyzed, with most having small sample sizes and significant results; all presented possible publication bias.

Classic fail-safe N for oximetric variables AHI and SO2 were estimated, whereby 2,821 and 87 studies respectively would be needed to counteract the results obtained in meta-analysis. This suggests that a considerable number of unpublished articles would be needed before the results of the present meta-analysis could be deemed insignificant.

For sleep variables, the classic fail-safe N for REM percentage was 45. But for AI and SE, with classic fail-safe Ns of 2 and 0, only a few non-significant studies would be needed to alter meta-analysis results, making these variables highly exposed to publication bias.

## Discussion

Although surgical treatment of SAHS through the removal of adenoids and tonsils (adenotonsillectomy) is considered the therapeutic option of choice, it has been noted that after this type of soft tissue surgery, some children continue to suffer respiratory problems (6). Given the multi-factorial etiology of SAHS, this could be due to untreated skeletal disorders (17). This theory has been supported by Guillemiault *et al.* (18) and Tasker *et al.* (19), who observed that some children treated with adenotonsillectomy developed SAHS as adults, leading the authors to suspect some skeletal basis for SAHS. Moreover, Katz *et al.* (20) report an association between maxillary compression and SAHS. For this reason RME could be an effective therapeutic option and studies such as Zhao *et al.* (21), using CBCT, have found that when maxillary compression is treated with RME, airway volume is enlarged in the nasal cavity and nasopharynx area (1) and the position of the tongue is improved contributing to increased space for the passage of air (1,21). So RME would appear to favor nasal respiration and could prevent airway obstruction (22). It has been shown that the outcomes of RME enjoy long-term stability (9,21). But a lack of space for the passage of air could also be due to oropharyngeal muscle hypotonia (22,23), and so research is underway into the efficacy of exercise-based coadjuvant therapies designed to achieve physiological respiration and to correct mouth breathing (23). Case/control studies would appear to indicate that myofunctional therapy reduces the residual symptoms remaining after adenotonsillectomy (23,12) and RME (12). Nevertheless, few studies have investigated this type of therapy, making it a line of research for future investigation.

Doubt remains as to how beneficial RME can be for treating SAHS in children who do not present maxillary compression, given the risks that accompany this therapy. In all the studies analyzed in the present literature review, all patients presented maxillary compression and it was for this reason that RME was applied. To date, no studies have been published on RME for treating SAHS in children without maxillary compression.

Two previous similar meta-analyses have been published (5,24) although the only variable analyzed was AHI. Both concluded that RME improved AHI values significantly, suggesting that RME is an effective treatment for SAHS. But Machado-júnior *et al.*, (24) included two studies with very similar samples in their meta-analysis (17,25), which could have weighted the qualitative synthesis, and so over-estimated the effect. To avoid this problem in the present meta-analysis, in three studies (13,17,25) analysis was only applied to one of the works, the one with the largest sample size (13), eliminating the other two to avoid any bias. The same problem occurred with samples in the studies Villa *et al.* (26) and Villa *et al.* (23), and so only the sample used in Villa *et al.* (23) was included for analysis, as this sample encompassed the sample used in Villa *et al.* (26), as explained in the latter article.

In the present meta-analysis, the studies included for analysis were disparate and did not follow similar methods or even the same treatment sequence, although they did analyze the same variables. Nevertheless, all the works obtained results that confirmed that RME is effective for treating SAHS in children (10,11,13,27). It would appear that RME improves nasal breathing (16), reduces nasal resistance, and mouth breathing disappeared in most of the children treated (22).

All the works obtained reductions in AHI to below 1, with the exception of two articles, but these works were found to be of poor quality (10,12). Miano *et al.* (10) registered an AHI that was higher than the range of values obtained in most of the studies, suggesting that a residual SAHS component remained after treatment. Guilleminault *et al.* (12) failed to obtain any considerable improvement in AHI values after the first treatment phase (regardless of whether this was RME or AM surgery) but after completing combined therapy SAHS was eliminated completely.

Villa *et al.* (11) argues that RME is a valid treatment option for children aged over 4 years with malocclusion and moderate SAHS, the age at which SAHS appears and its severity being the main factors influencing the choice of treatment. The authors confirmed that SAHS was resolved in most cases. Pirelli *et al.* (13) stressed that RME is a valid treatment option providing the child does not present adenoidal and tonsil hypertrophy. Nevertheless, SAHS is produced by an interaction between the skeletal component and soft tissue enlargement (12). In these cases, RME can act as a coadjuvant therapy after surgery (16). The work by Guilleminault *et al.* (12) appears to confirm this, obtaining an improvement in all signs and symptoms after applying RME and surgery in combination, regardless of the order in which they were carried out. They report that in some cases the SAHS was resolved with the first treatment phase, although they do not propose any criteria to determine which treatment to apply first. In light of these findings, future research should set out to establish protocols to determine the predominant disorder in order to decide which treatment to apply first; thereafter, if the signs and symptoms do not disappear, an additional therapy can de applied.

Lastly, RME could have an important role to play in preventative treatment of SAHS in children (22). Early diagnosis is important to prevent the consequences of the syndrome (1).

The present review suffered some limitations, in particular, the varying methods used in the articles analyzed, and the lack of high quality randomized case/control studies. In addition, as outlined above, some susceptibility to publication bias was detected. But in spite of these limitations, most of the works reviewed showed that RME would appear effective for treating slight or moderate cases of SAHS in children, as most of the variables measured underwent significant improvements. The level of evidence was acceptable, although the review points to a need to establish measurement protocols that would allow clearer data comparison.
